# Differences in growth rates and pre-hibernation body mass gain between early and late-born juvenile garden dormice

**DOI:** 10.1007/s00360-016-1017-x

**Published:** 2016-08-01

**Authors:** Stefan Stumpfel, Claudia Bieber, Stéphane Blanc, Thomas Ruf, Sylvain Giroud

**Affiliations:** 1 Department of Integrative Biology and Evolution, Research Institute of Wildlife Ecology, University of Veterinary Medicine, Vienna, Savoyenstraße 1, 1160 Vienna, Austria; 2 Université de Strasbourg, IPHC, 23 rue Becquerel, 67087 Strasbourg, France; 3 CNRS, UMR7178, 67087 Strasbourg, France

**Keywords:** *Eliomys quercinus*, Hibernation, Body length, Fattening, Maternal care

## Abstract

Juvenile hibernators have to allocate energy to both growth and fattening, to survive winter, and to avoid possible disadvantages during their first reproductive season. Being born late in the active season may have important effects on growth and fattening. This study aimed at determining potential differences in rates and maximal level of growth, and in pre-hibernation body fat mass between early and late-born juvenile garden dormice (*Eliomys quercinus*), and kept in outdoor enclosures with ad libitum food and water. We first assessed mean pup mass in early and late-born litters (*n* = 31) from birth to their early weaning phase, at which time body composition was determined. Then, growth and body mass of early and late-born individuals (six males and six females, for each group) were measured weekly until hibernation onset (*n* = 24). We also assessed fat content in a group of juveniles during pre-hibernation fattening (*n* = 16) and after their first winter hibernation (*n* = 18). During the pre-weaning phase, young from early and late litters mainly grew structurally and gained mass at similar rates. After weaning, late-born juveniles grew and gained mass twice as fast as early born individuals. Body mass was positively associated with fat content during pre-hibernation fattening. Late-born females reached similar structural sizes, but had lower pre-hibernation fat reserves than early born females. Conversely, late-born males showed lower maximal size and pre-hibernation body fat content, compared with early born males. Thus, individuals born late in the season cannot fully compensate the lack of available time before the winter onset.

## Introduction

To survive the winter season, many small mammals enter hibernation, a state of reduced metabolic rate and body temperature, and rely mainly on their body fat stores throughout winter (for review see Dark 2005). Hence, gaining sufficient body fat reserves in autumn to last throughout hibernation is an important process (Phillips 1981; Dark et al. 1992; Mugaas et al. 1993). Indeed, pre-hibernation body mass (BM), which is a good indicator of fat content (Schaefer et al. 1976), correlates positively with overwintering survival (Armitage et al. 1976; Rieger 1996). This seems especially true for juveniles due to their lower fat reserves prior to hibernation (Dark 2005). Indeed, obtaining sufficient fat stores is particularly difficult for juveniles, especially for those born late in the season of reproduction, due to the limited time available for growth and fat accumulation prior to their first hibernation season (Lenihan and van Vuren 1996). For instance, juveniles born when the summer season is well advanced have less time to grow and to gain sufficient BM for hibernation compared to conspecifics born earlier in the season (Pilastro et al. 1994; Dmitriew 2011). Armitage et al. (1976) found that juvenile yellow-bellied marmots (*Marmota flaviventris*) weaned earlier are more likely to survive hibernation. Similar results were found by Blumstein and Arnold (1998), and Rieger (1996). Rieger showed that a variation of about 20 days between weaning times of first and last litters of Uinta ground squirrels [*Spermophilus armatus* (=*Urocitellus armatus*)] had a significant negative effect on winter survival.

To compensate for the lack of available time, late-born juveniles would have to grow and to fatten faster than early born individuals to reach sufficient levels of body size and body fat mass (FM). It has been reported that late-born juvenile European hedgehogs (*Erinaceus europaeus*) gained BM significantly faster than early born individuals (Bunnell 2009). In addition, there seem to be sex differences in maximal levels of body mass prior to hibernation in juveniles from species that are sexually dimorphic. For instance, in European ground squirrels (*Spermophilus citellus*), male juveniles reached a higher maximum BM than female juveniles (Millesi et al. 1999). However, to date, we lack detailed studies investigating differences in development between juvenile hibernators born at different times within the active season.

This study aimed to determine growth rates, BM gain and pre-hibernation levels of body size and fat reserves as a function of the time of birth (early vs. late-born) and sex, in juvenile garden dormice. We expected late-born individuals (1) to display faster growth rates and BM gains prior to winter, (2) and/or to possibly delay hibernation onset, and hence (3) to reach similar levels of body size and BM prior to hibernation, compared with their early born counterparts. We also hypothesized that in this species, BM is closely correlated with, and can be used as a proxy of, FM prior to hibernation.

## Materials and methods

### Animals and experimental conditions

The garden dormouse is a small omnivorous, nocturnal rodent widely distributed in Western Europe. This species inhabits mainly coniferous and mixed forests and is found up to 2000 m elevation in the Alps (Amori et al. 1994). The garden dormouse shows variation in the duration of its hibernation period. Animals from the Jura Mountains (1400 m) hibernate for at least 8 months, generally from mid-September to June, but in Mediterranean areas, they only hibernate for 1–3 months (Vogel 1997; Gil-Delgado et al. 2006). Both in the field and in captivity, the garden dormouse can have two litters during a reproductive season, one in late spring (May–June) and another one in late summer (August) (Moreno 1988; Bertolino et al. 2001; Giroud et al. 2012, 2014).

All animals of this study were born in a breeding colony at the Research Institute of Wildlife Ecology (FIWI, University of Veterinary Medicine Vienna, Austria; latitude 48°15′N, longitude 16°22′E) from a stock originally captured in Western Germany (‘Ebertseifen’, Niederfischbach, Germany). In this study, five experimental groups were included (see Table 1) with 24 juveniles in the main experiment (‘main experiment’; see below for more details), 18 individuals for determining fat content during pre-hibernation fattening and after hibernation (‘fattening’; see below for more details), 193 pups from 31 l (‘early weaning’ and ‘pre-weaning’; see below for rationale), and 10 adult garden dormice (‘adults’, see below for rationale).

**Table 1:** Experimental groups including in the study

Dataset	Description	Size
‘Main experiment’	Comparison of growth rates, body mass gains and pre-hibernation levels of body size and body mass in early vs. late-born male and female juveniles	*N* = 24 animals (6 per group*)
‘Fattening’	Determination of fat content of juveniles during their pre-hibernation fattening phase and after hibernation (same individuals)	*N* = 18 animals
‘Early weaning’	Determination of body composition of litters around the early post-weaning phase	*N* = 30 l
‘Pre-weaning’	Comparison of growth rate in early vs. late-born litters during the pre-weaning period	*N* = 31 l (23 EB, 8 LB)
‘Adults’	Comparison of pre-hibernation body size levels of juveniles from the ‘Main Experiment’ with those of adult male and female garden dormice	*N* = 10 animals (5 M, 5 F)

* Groups are early born (‘EB’) and late-born (‘LB’) male (‘M’) and female (‘F’) juvenile garden dormice

The experimental group ‘main experiment’ (Table 1) included 12 early born juveniles (six males and six females) born in mid/late May and 12 late-born juveniles (six males and six females) born in early August 2013. Early and late-born juveniles had the same mothers and originated from four different litters. By regularly weighing the offspring, we choose to separate them all from the mothers at a similar BM of 40.1 ± 3.6 g to start the experiments. At this stage, juvenile dormice were independent from their mother and could already sustain their own development by relying on solid food. In our colony, young garden dormice start to eat solid food, on average, at an age of 30 days (S.G., personal observation). We subsequently refer to this period as ‘early weaning phase’.

Animals were then housed in groups of 6 individuals in outside enclosures divided by sex and group (early vs. late-born). Each enclosure was provided with small branches and three straw-filled wooden nest boxes. All animals were maintained under natural variations of photoperiod and ambient temperature. Near the enclosures, a temperature logger (EL-USB2, Lascar Electronics, Salisbury, UK, accuracy ± 0.5 °C) recorded the ambient temperature in the shade every half hour. All juveniles were provided with *ad* *libitum* access to food and water. Food consisted of dry cat pellets (Topix, Saturn Petfood, GmbH, Bremen, Germany), rodent chow (Altromin 7024 Spezialfutter, GmbH & Co. KG, Lage, Germany), and sunflower seeds.

To validate the use of BM as a proxy for pre-hibernation fat content, body FM was assessed in a group of 18 late-born juveniles (Table 1, ‘fattening’) during their pre-hibernation fattening phase, i.e., at an average BM of 82.3 ± 6.7 g (see section “body composition” below). At that time of the years, animals had not yet reached their maximal pre-hibernation body mass (92.8 ± 0.9, see Fig. 4) and were still gaining body mass prior to hibernation. The same 18 individuals were used to determine body FM immediately after terminating their first winter hibernation without feeding (BM = 61.1 ± 9.6 g, see section “body composition” below). Furthermore, body composition was also measured in 30 additional litters (Table 1, ‘early weaning’), 35.7 ± 3.7 days after birth, i.e., around the early weaning phase (see section “body composition” below).

To further investigate potential differences in pre-weaning growth rate between early and late-born litters, we assessed litter size at birth, and measured litter mass gain in 23 early born and 8 late-born litters (Table 1, ‘pre-weaning’). Measurements were performed at 3, 6, and 9 days after birth, and then weekly for all litters until 35 days post-birth.

### Body growth and body mass measurements

Structural growth and BM were assessed in the 24 juveniles of the ‘main experiment’ (Table 1) once a week (in the morning) from the time of separation from the mothers until the start of hibernation. For each group, the first week of measurements was defined as week 0. Hibernation onset was defined as the time when all of the juveniles did not feed anymore, as determined by weighing provided and remaining food (data not shown).

Each animal or litter was weighed to the nearest 0.1 g (Mettler Toledo, PM34, Delta Range). We assessed structural growth by measuring individual body length (BL), i.e., the distance from the tip of the nose to the base of the tail, which represents the best estimate of growth in garden dormice (data not shown). For the measurement of growth, the same person measured BL of each animal three times (to the nearest mm) with a tape measure (Hoechstmass-Balzer GmbH, Sulzbach, Germany). The length of each individual was computed from the mean of three measurements. Single measurements of BL for early born male juveniles of week 1 were excluded because of a too large measurement error.

To determine whether juvenile dormice reached adult body size prior to their first winter, we also measured the body length of ten adult individuals (five females and five males; Table 1, ‘adults’), kept in our enclosures, with an age of 2 years in fall (October). The same individuals were measured again at 4 years of age.

### Body composition measurements

Body composition was determined in 18 juveniles (Table 1, ‘fattening’) during pre-hibernation fattening in fall by isotope dilution during a protocol using the doubly labeled water (DLW) method to assess total energy expenditure, as already used in garden dormice previously (for details on the methodology, see Giroud et al. 2014). The DLW method for determining body composition during the pre-hibernation fattening was used to allow the animals to subsequently enter winter hibernation. For each individual, a baseline urine sample was taken by gently stimulating urination. Each animal was injected intraperitoneally a premixed 5 g/kg dose of DLW, thinned with 3 % NaCl to physiological osmolarity. Isotopic equilibration in body water was determined from a blood sample collected at 1-h post-dose from a quick sampling of the saphenous vein. Approximately 300 µl of blood, representing less than 0.5 % of the animal BM, was taken from each individual (BM ~80 g). The blood was collected in micro-capillary tubes, which were immediately flame-sealed. Blood capillaries were stored at 4 °C, and urine samples were kept frozen at −20 °C until subsequent analyses by isotope ratio mass spectrometry, at the Department of Ecology, Physiology and Ethology (IPHC, CNRS-UdS, Strasbourg, France), as previously described (Chery et al. 2015). The total body water was derived from this time point by the principle of isotopic dilution. Fat-free (lean) mass was calculated assuming a hydration coefficient of 73.2 %, which has been reported to be very stable across species (Schoeller 1996), and FM was computed by difference with BM. Due to leaks in micro-capillary tubes, blood samples from two individuals could not be analyzed, hence reducing the sample size from 18 to 16 animals.

At early weaning phase and after the first winter hibernation, body fat content was assessed in 30 additional litters (Table 1, ‘early weaning’) and in 18 juveniles (Table 1, ‘fattening’), respectively, using the Soxhlet method (Soxhlet 1879). Animals were killed by carbon dioxide euthanasia, and dead bodies were weighed, minced, and rubbed in sea sand. Afterward, they were dried in a heat cabinet at 103 °C for 48 h and then cooled in an exsiccator. Fat was extracted by repeatedly washing with petroleum ether, under reflux in a glass flask for 6 h. Both fat content and lean mass were expressed in grams.

### Statistics

All statistical computations were performed by using R 3.0.2 (R Development Core Team 2013). Residuals from statistical models were inspected for normality using quantile–quantile plots and histograms of distributions, and if necessary, response variables were Box–Cox transformed to achieve normality. Means are given ±SD and regression coefficients ±SE. *p* < 0.05 was considered significant.

To assess group (early vs. late-born) and sex effects on BL and BM at the start of the experiment, we used linear models. From the repeated measurements of individual BL and BM over time (i.e., weeks), segmented regressions (with ambient temperature as fixed factor) were used to determine three response variables, namely, the rate (‘slope’) of structural growth (i.e., BL) or BM gain, the maximal level (‘plateau’) at which structural growth (i.e., BL) or BM plateaued, the time (‘time of plateau’) at which the plateau of structural growth (i.e., BL) or BM occurred. Segmented regressions were constituted of the combination of two linear regressions (linear models), one on the first linear increase in BL or BM over time, and one on the following linear part, corresponding to the ‘plateau’. The inflection point (‘time of plateau’) between the two linear parts was defined as the time-point when BL or BM increased by less than 2 %. For each of the three response variables, the full linear mixed effects model included sex, group, and the group:sex interaction, as well as individual ID as random factor. We subsequently computed all possible reduced models and used model averaging to determine model-average coefficients [package ‘MuMIn’ (Bartoń 2012)]. We also computed the relative importance of the predictor variables (RVI) (including interactions) from a sum of the Akaike weights over a set of supported models (ΔAIC <10) in which the term of interest appeared. We further applied a Tukey-like post hoc multiple comparison test [R package ‘multcomp’ (Hothorn et al. 2008)] to reveal specific differences in maximal BL (i.e., at the ‘plateau’) between early born and late-born males and females.

We used ranged major axis (RMA) models (R package ‘lmodel2′ (Sokal and Rohlf 1995; Legendre and Legendre 1998) to test for significant associations between FM and BM both during pre-hibernation fattening and at post-hibernation. For the early weaning phase, we computed means of pup body, lean, and fat masses for each litter, by dividing either litter mass, lean mass or FM by the respective litter size. Then, linear models were used to test for significant associations between mean pup fat or lean mass and mean pup mass. Similarly, mean pup body masses were calculated from masses of early and late-born litters over 35 days after birth, and tested for significant effects of being born early or late using a linear model. Finally, we used linear models to test for possible differences in litter size at birth between early and late-born litters, as well as in BL between 2-year-old and 4-year-old male or female garden dormice.

## Results

### From birth to the early weaning phase

**Fig. 1:**
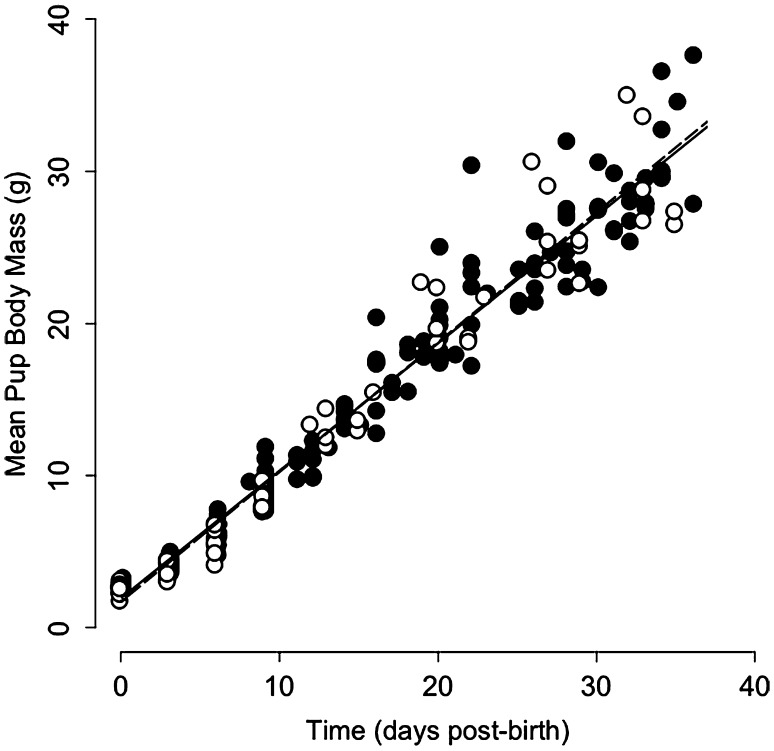
Mean body mass of pups from early born (*black dots*, *n* = 23) and late-born litters (*white circles*, *n* = 8) during the first 35 days after birth. Mass of each of the 30 l was assessed at 3, 6, and 9 days after birth, and then weekly for all litters. Note that slopes of regression lines from early born (*continuous line*) and late-born litters (*dashed line*) did not significantly differ from each other (*t* = 0.70, *p* = 0.49)

Among the 31 l (Table 1, ‘pre-weaning’) neither the mass nor litter size at birth differed between early and late-born litters (mass: 16.9 ± 3.6 vs. 16.7 ± 3.8 g, *t* = −0.16 *p* = 0.87, Fig. 1; size: 6.2 ± 1.3 vs. 6.4 ± 1.2 pups, *t* = 0.38, *p* = 0.70). During the 35 days after birth, pups significantly gained BM at an average rate (derived from weighing 31 l) of 0.84 g day^−1^ (*t* = 71.71, *p* < 0.001, adjusted *R* ^2^ = 0.96; Fig. 1). However, mean pup BM gain did not differ between early and late-born litters over this period (*t* = 0.70, *p* = 0.49; Fig. 1).

Among the 24 juveniles included in the main experiment (Table 1, ‘main experiment’), sizes of early and late litters were 5.0 ± 0.8 and 5.3 ± 1.3 pups, respectively, and did not differ from each other (*t* = 0.33, *p* = 0.75). Late-born and early born individuals were separated at a similar age from the mothers, i.e., 40.0 ± 1.0 and 42.2 ± 6.5 days after birth, respectively (*F* = 1.29, *p* = 0.27).

### From early weaning phase to the onset of hibernation

#### Body size

**Fig. 2:**
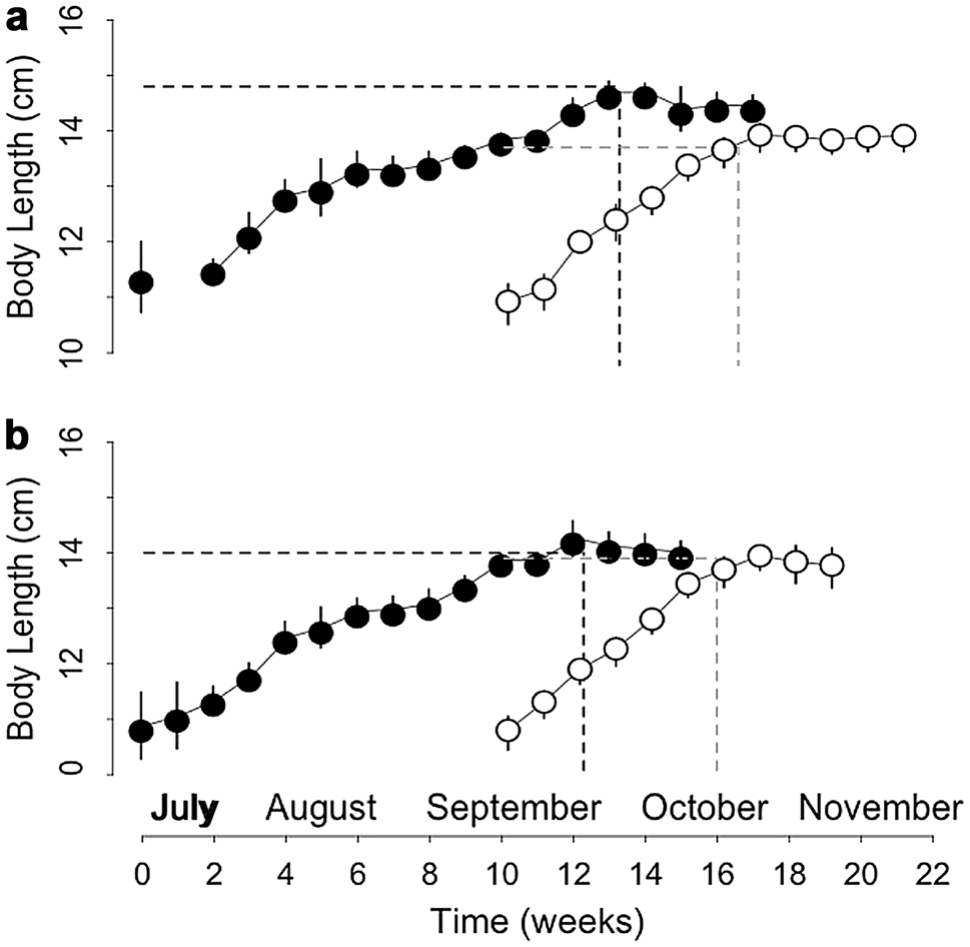
Changes in structural growth (body length) of **a** male juvenile and **b** female juvenile garden dormice, born either early (*black circles*) or late (*white circles*), from the start of experiments until hibernation onset. The *dotted lines* indicate the maximal level (‘plateau’), at which body length leveled-off, and the time (‘time of plateau’) at which the plateau of body length occurred. *N* = 6 individuals per group and values are mean ± SD

At the start of the experiments, BL of the 24 juveniles (Table 1, ‘main experiment’) did not significantly differ between sexes and groups (group: *t* = −0.62, *p* = 0.55; sex: *t* = 1.62, *p* = 0.12; Fig. 2).

**Fig. 3:**
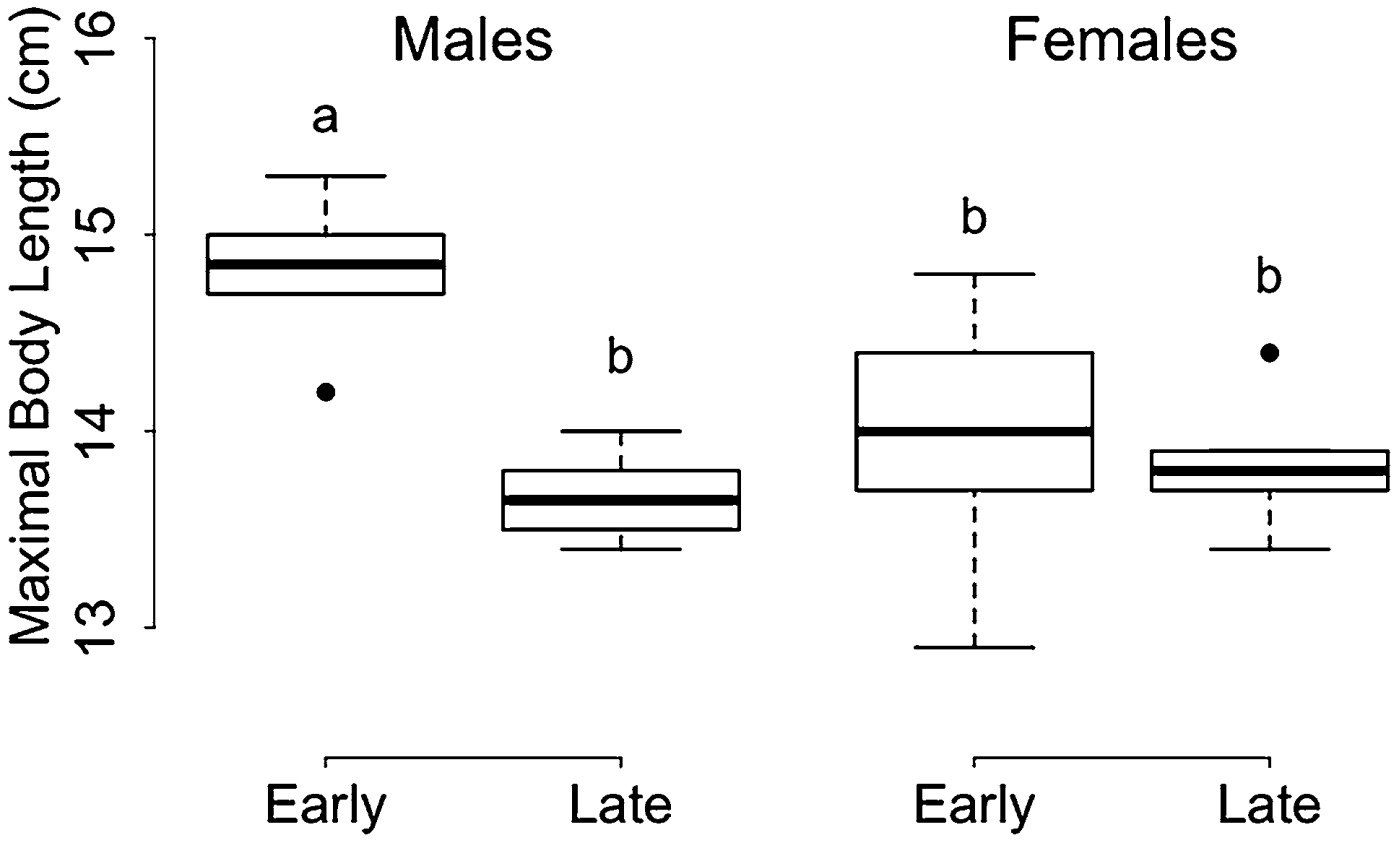
Maximal body size (body length) of early (‘early’) and late-born (‘late’) males (*left panel*) and females (*right panel*) prior to hibernation, i.e., at the ‘plateau’. Groups (*n* = 6 individuals per group) differing significantly (*p* < 0.01, Tukey’s post hoc comparisons) are denoted by different *superscripts*

The slope of BL increase differed significantly between groups, with late-born juveniles growing at a 1.6-fold faster rate than early born individuals (Fig. 2; Table 2). The BL plateau differed between early and late-born males (Fig. 2a), but not between early and late-born females (Fig. 2b), as indicated by the significant interaction between group and sex (Table 2). Indeed, post hoc analyses revealed that only early born males reached a significantly higher maximal body size prior to hibernation, compared with all three other groups (Fig. 3). Significant differences in the time of plateau were also detected between both sexes and groups (Table 2; Fig. 2). Early born males reached a maximum BL of 14.8 ± 0.4 cm at week 13.2 ± 0.4, whereas late-born males reached a lower maximum BL of 13.7 ± 0.2 cm, but only 6.5 ± 0.6 weeks after their separation from the mothers (Fig. 2a; Table 2). Early born females reached a maximum BL of 14.0 ± 0.7 cm at week 12.2 ± 0.4, whereas late-born females reached a similar maximum BL level of 13.9 ± 0.3 cm, already 5.9 ± 1 weeks after separation from the mothers (Fig. 2b; Table 2).

**Table 2:** Averaged model parameters for the effects of group (early born vs. late-born, ‘LB’) and sex on the rate (‘Slope’) of body mass (‘BM’) gain and structural growth (assessed by body length, ‘BL’), the maximal level (‘Plateau’) at which BM or BL leveled off and the time (‘time of plateau’) at which the plateau of BM or BL occurred during the pre-hibernation period

Parameter	Response variable	Term	RVI	Coefficient	Adjusted SE	*Z* value	*p* value
BM	Slope	Group (LB)	1.00	4.31	0.51	8.46	**<0.001**
		Sex (male)	0.41	−0.24	0.58	0.41	0.68
		Group:sex	0.15	−1.11	0.87	1.28	0.20
	Plateau	Group (LB)	0.97	−14.72	6.60	2.23	**<0.05**
		Sex (male)	0.43	8.71	7.44	1.17	0.24
		Group:sex	0.18	−15.35	10.55	1.46	0.15
	Time of plateau	Group (LB)	1.00	−5.85	0.39	14.95	**<0.001**
		Sex (male)	1.00	1.78	0.39	4.54	**<0.001**
		Group:sex	0.17	−0.20	0.73	0.73	0.78
BL	Slope	Group (LB)	1.00	0.22	0.03	6.99	**<0.001**
		Sex (male)	0.98	−0.04	0.03	1.21	0.23
		Group:sex	0.59	−0.07	0.04	1.81	0.07
	Plateau	Group (LB)	1.00	−0.19	0.32	0.60	0.55
		Sex (male)	0.94	0.83	0.30	2.79	<0.01
		Group:sex	0.87	−1.03	0.38	2.69	**<0.01**
	Time of plateau	Group (LB)	1.00	−6.59	0.39	17.06	**<0.001**
		Sex (male)	1.00	1.16	0.39	3.01	**<0.01**
		Group:sex	0.57	−0.88	0.50	1.75	0.08

*RVI* relative variable importance

*p* values shown in bold correspond to statistically significant and interpretable values

BL did not differ between 2-year-old and 4-year-old males (16.2 ± 0.5 vs. 15.7 ± 0.4 cm, *t* = −1.82, *p* = 0.11) or females (15.4 ± 0.4 vs. 14.9 ± 0.4 cm, *t* = −2.27, *p* = 0.06). Both early and late-born juveniles had a significantly lower maximum BL compared with 2-year-old adult individuals (Table 3). Body size also significantly differed between adult males and adult females (16.2 ± 0.5 vs. 15.4 ± 0.4 cm, *t* = 2.88, *p* < 0.05).

**Table 3:** Comparison of maximal levels of body size (body length) between early or late-born juveniles and 2-year-old adult garden dormice, prior to winter hibernation

Sex	Early born	Late-born	Adult	Statistics	
				Early born vs. adult	Late-born vs. adult
Males	14.8 ± 0.4 cm (*n* = 5)	13.7 ± 0.2 cm (*n* = 6)	16.2 ± 0.5 cm (*n* = 6)	*t* = −6.97, ***p*** **<** **0.001**	*t* = −11.22, ***p*** **<** **0.001**
Females	14.0 ± 0.7 cm (*n* = 5)	13.9 ± 0.3 cm (*n* = 6)	15.4 ± 0.4 cm (*n* = 6)	*t* = −6.31, ***p*** **<** **0.001**	*t* = −8.45, ***p*** **<** **0.001**

*p* values shown in bold correspond to statistically significant values

#### Body mass^1^

**Fig. 4:**
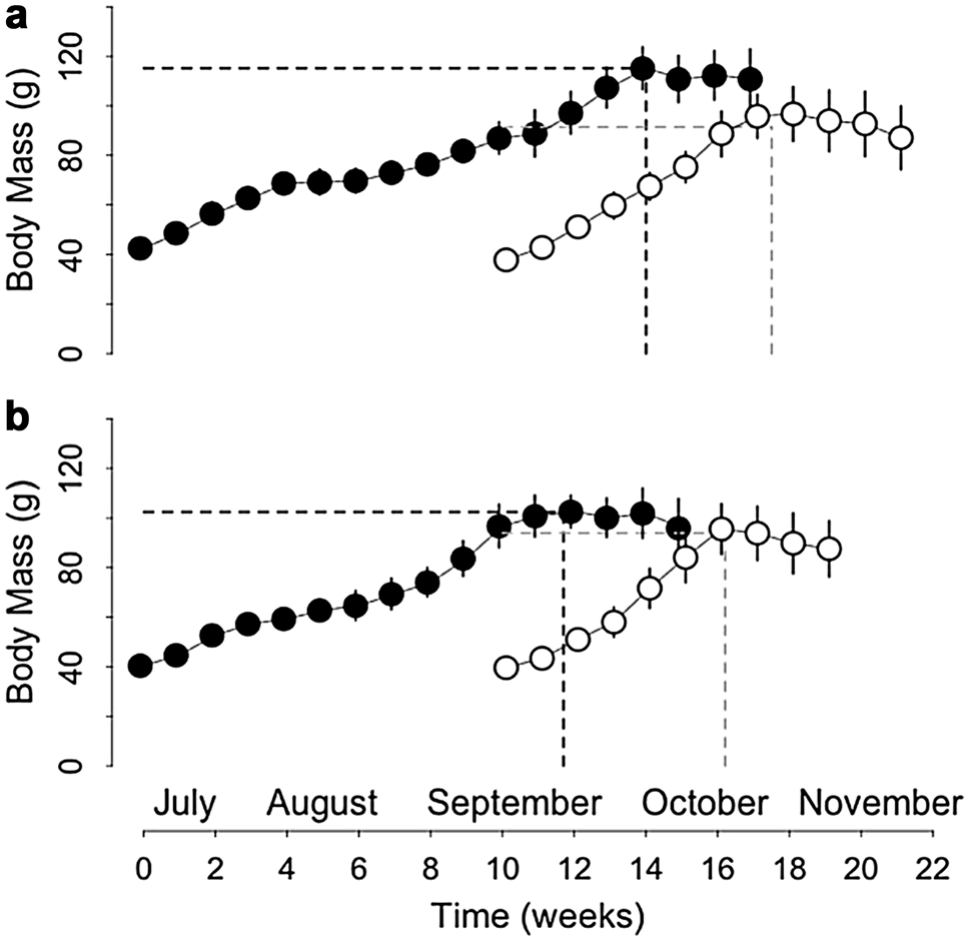
Body mass gain of **a** male juvenile and **b** female juvenile garden dormice, born either early (*black circles*) or late (*white circles*), from the start of experiments until hibernation onset. The *dotted lines* indicate the maximal level (‘plateau’), at which body mass leveled off, and the time (‘time of plateau’) at which the plateau of body mass occurred. *N* = 6 individuals per group and values are mean ± SD

BM of the 24 juveniles (Table 1, ‘main experiment’) did not differ between groups and sexes at the start of the experiment (group: *t* = −0.51, *p* = 0.62; sex: *t* = 1.12, *p* = 0.28; Fig. 4).

The slope and plateau of BM significantly differed between groups, but not between sexes (Fig. 4; Table 2). Late-born juveniles gained mass 1.9-fold faster and reached a 15 % lower maximum BM level compared with early born individuals (92.8 ± 0.9 vs. 108.8 ± 1.1 g; Fig. 4).

Significant differences in the time of plateau were detected between sexes and groups (Fig. 4; Table 2). Females reached their maximum BM 1.7 week earlier than males (9.8 ± 0.8 vs. 11.5 ± 1.0 weeks after their separation from the mothers; Fig. 4). Similarly, late-born individuals reached their maximum BM 7.3 weeks earlier than their early born counterparts (7.0 ± 0.6 vs. 14.3 ± 1.2 weeks after their separation from the mothers; Fig. 4).

#### Body composition assessed by Soxhlet and DLW

At the early weaning phase (Table 1, ‘early weaning’), mean mass of pups was 27.9 ± 3.1 g. No correlation was found between the mean mass and mean fat content of the pups (*t* = −1.06, *n* = 30, *p* = 0.30). FM representing only 5.9 ± 2.1 % of the BM of pups, lean mass accounted nearly for the totality of pup BM, hence, was strongly positively associated with total BM (*t* = 64.94, *n* = 30, adjusted *R* ^2^ = 0.99, *p* < 0.001).

**Fig. 5:**
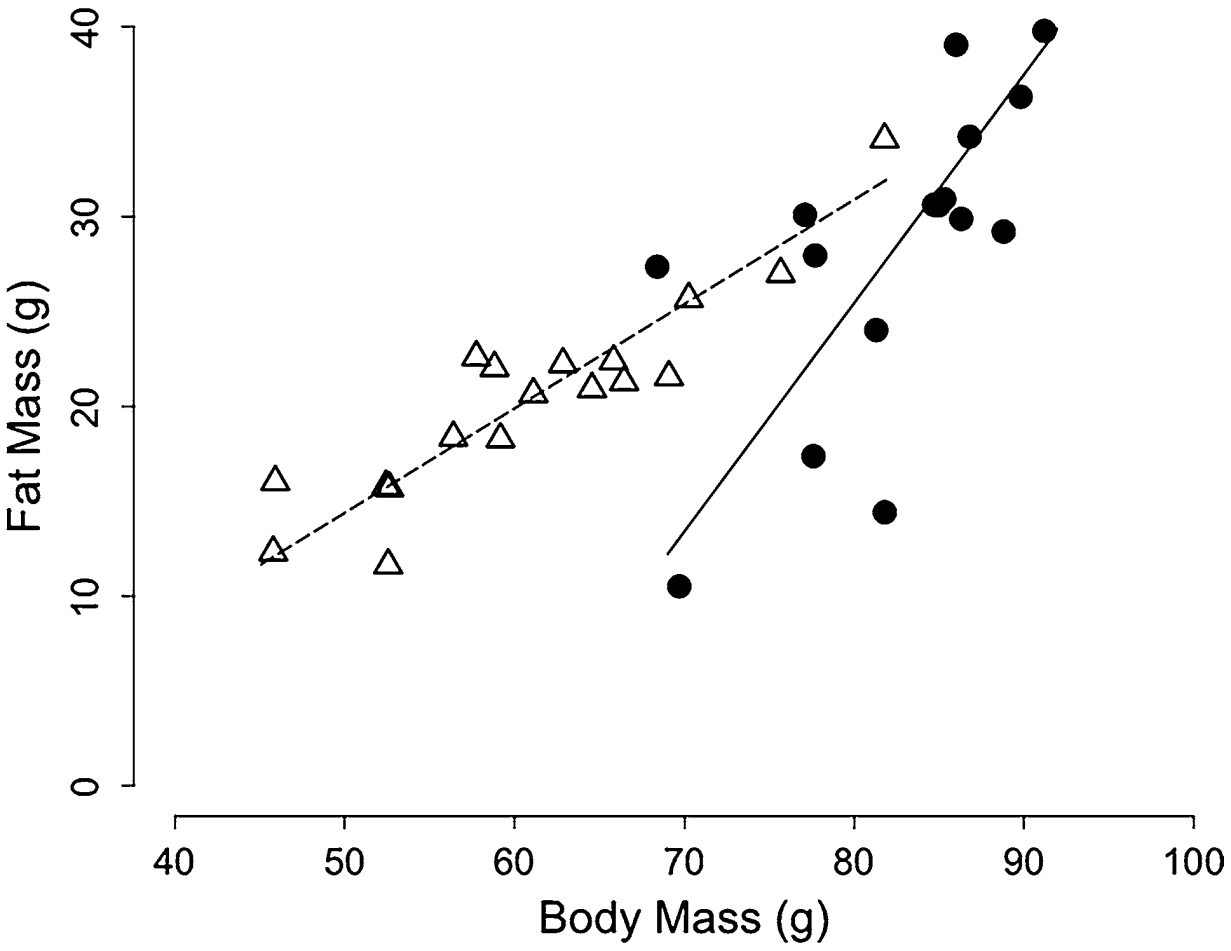
Fat mass as a function of body mass in juvenile garden dormice during the pre-hibernation fattening phase (*black dots*, *n* = 18) and after winter hibernation (*white triangles*, *n* = 16). The same individuals were measured before and after hibernation (see Table 1 and the method’s section ‘Animals and experimental conditions’ for more details). Note the significant difference of slopes between groups (pre-hibernation fattening vs. after hibernation)

We found significant positive relationships between BM and fat content across individuals (Table 1, ‘fattening’), both during pre-hibernation fattening (intercept = −70.86, slope = 1.20 ± 0.09, adjusted *R* ^2^ = 0.45, *n* = 16, *p* = 0.005; Fig. 5) and after winter hibernation (intercept = −13.13, slope = 0.55 ± 0.01, adjusted *R* ^2^ = 0.83, *p* = 0.001; Fig. 5). Furthermore, the slope of the relationship was significantly steeper during pre-hibernation fattening vs. after hibernation (1.20 ± 0.09 vs. 0.55 ± 0.01 CI), indicating that individual differences in BM were due to differences in fat content to a high degree during pre-hibernation fattening and to a lower degree after hibernation.

## Discussion

### Differences in growth rate and maximal body size between early and late-born juveniles

Our results show that late-born juvenile garden dormice gain BM much faster (1.7-fold for males and 1.9-fold for females) than early born individuals prior to their first winter hibernation. These results are in line with findings of Bunnell (2009), reporting that late-born European hedgehogs gained weight significantly faster than early born individuals leading to improved chances of overwinter survival. Conclusions of this study were, however, only based on BM changes and could not discriminate between growth and fattening processes. Our study demonstrates that late-born juvenile garden dormice, both males and females, grow structurally faster compared with individuals from early litters. Moreover, this growth acceleration occurs predominantly during the post-weaning period, since no compensation in BM gain occurs in late-born litters during lactation. Potential mechanisms for a fast body mass gain may be linked to increased food intake and/or reduced activity (Körtner and Geiser 1995). For instance, highest rates of weigh gain were observed in peak activity season in marmots (Young 1984; Körtner 1991). In contrast, mountain pygmy possums (*Burramys parvus*) were reported to decrease their daily activity levels during the pre-hibernation fattening period. Similar observations were made in ground squirrels (*Spermophilus tridecemlineatus* [=*Ictidomys tridecemlineatus*]), suggesting that pre-hibernation body mass gain occurs during a time of reduced activity and thus low energy expenditure (Mrosovsky 1971). Recently, we have also found that late-born garden dormice use short bouts of torpor, i.e., a state of reduced metabolic rate, to sustain body mass gain prior to hibernation (Giroud et al. 2014). However, the exact underlying mechanisms leading to different growth rates and body mass gains in early and late-born juvenile garden dormice remain to be determined.

Our study further demonstrated that late-born males reach a lower maximal body size prior to hibernation, compared to their early born counterparts. A reduced body size, hence BM, could have disadvantages for males in the following reproductive season, especially in terms of male–male competition, access to favorable territories and subsequent reproductive success. Indeed, it has been described that, at emergence from hibernation, male garden dormice form groups with hierarchical structures, and the weakest or smallest animals have a reduced access to females and are forced to disperse (Vaterlaus-Schlegel 1997). However, the comparison of body sizes between adult and juvenile garden dormice prior to hibernation showed that both early and late-born individuals did not complete their structural growth before their first winter. Thus, both early and late-born sub-adult males might not be able to compete with adult males for reproduction after their first hibernation season. However, being of larger body size prior to winter and, hence, at emergence from hibernation can be seen as an advantage for early born males to reach an adult body size earlier and to be able to compete at an earlier stage with adult individuals.

The observed growth patterns are common for mammalian species confronted with marked seasonal environments, in which young individuals grow during a longer period, compared with those from non-seasonal species (Morton et al. 1974). Indeed, Belding ground squirrels (*Spermophilus beldingi* [=*Urocitellus beldingi*]) do not achieve their maximum body size until the end of the second season of their life (Morton and Tung 1971). Fietz and Weis-Dootz (2012) also reported that growth occurred until at least the age of 4 years in edible dormice. In this study, 2- and 4-year-old individuals showed no difference in their body size, suggesting that garden dormice reach their adult size within the first 2 years, similar to Belding ground squirrels. Furthermore, there seems to be a sexual dimorphism in garden dormice, adult males being slightly (~5 %) but significantly larger than adult females. This sex difference in body size might explain why female juveniles, especially those born early, even with sufficient time before winter start, do not grow to a level similar to early born males prior to hibernation.

Being born late might lead to a trade-off between costs and benefits of growing at a normal rate, i.e., delaying the time of reaching an adult body size, and growing at a maximal rate (Metcalfe and Monaghan 2003). Indeed, there is increasing evidence that fast growth may cause long-term negative effects during adulthood, such as suboptimal overall performance with lower reproductive success, accelerated aging and reduced lifespan (Metcalfe and Monaghan 2003). For instance, strains of laboratory mice and rats with more rapid juvenile growth tend to have reduced adult lifespans (Bartke et al. 2001; Rollo 2002). Similar effects have also been described in fish (Comfort 1963) and in wild lizards (Olsson and Shine 2002). Compensatory growth, i.e., accelerated growth after a period of poor nutrition, has also been found to have carryover effects into adulthood (see Metcalfe and Monaghan 2003; Monaghan 2008). In particular, compensatory growth can be linked to oxidative stress damage, due to reactive oxygen species, on key biomolecules, like DNA, proteins, and lipids (Finkel and Holbrook 2000). It has been reported that in damselflies, after a period of reduced mass gain, compensatory growth in the larval stage was associated with higher oxidative stress in the adult stage (De Block and Stoks 2008). In addition, red blood cell resistance to free radicals was negatively correlated with growth rate in zebra finches (Alonso-Alvarez et al. 2007). This relation was only significant during the period of accelerated growth, suggesting that fast or compensatory growth can negatively affect resistance to oxidative stress of the individual (Alonso-Alvarez et al. 2007). On the other hand, being smaller usually means being more vulnerable and less competitive in terms of access to food and reproduction (Arendt 1997). In additon, a fast growth normally requires increased food intake, hence higher foraging activity, which in turn may expose individuals to a greater risk of being caught by a predator (Gotthard 2000). Despite potential costs of growing fast, there is clearly an advantage to escaping from vulnerable early life and sub-adult stages as quickly as possible by attaining a larger body size. Furthermore, rapid growth can provide other advantages, even if the maximal lifespan is reduced. For instance, the maximal amount of energy reserves that can be stored increases with body size; therefore, larger individuals have a greater resistance to food deprivation (Ludsin and DeVries 1997). Moreover, heat loss will be lower in larger animals, which will hence expend less energy per gram body mass. However, individuals of larger body size will have higher total energy expenditure during arousals (which, in turn, may be compensated by the ability to store more fat). In the context of this study, increased growth rates in late-born juveniles would allow them to store sufficient fat reserves to survive the subsequent winter hibernation.

### Pre-hibernation levels of fattening

Fat content of juvenile garden dormice was positively associated with their BM both during pre-hibernation fattening and after their first winter hibernation (without food). Interestingly, the slope of this relationship during pre-hibernation fattening was significantly higher than after hibernation. These results indicate that in fall, BM gain was completely (slope ~1) due to fat deposition, whereas in spring, differences in BM were equally (slope ~0.5) due to variation in fat and lean mass. In the current context, it is important to note that, during the pre-hibernation phase, BM of juvenile dormice indeed represents an excellent proxy for body fat content. A similar conclusion has been inferred from results on edible dormice, *Glis glis* (Schaefer et al. 1976). Indeed, BM fluctuations during the fattening process in edible dormice are exclusively caused by variations of their body FM with the lean mass of the animals remaining almost constant throughout the BM cycle (Schaefer et al. 1976).

In this study, late-born juveniles reached a 15 % lower maximal BM, and hence body fat content, than early born individuals. A lower pre-hibernation fat content is thought to decrease chances of individual overwintering survival and potentially the reproductive success in the following year (Arnold 1990; Pilastro et al. 1994). Among late-born males, the lower BM is partially explained by a 9 % lower body size, but also by a lower amount of fat content. Hence, juvenile garden dormice did not reach pre-hibernation fat reserves similar to early born individuals, contrary to juveniles of Belding ground squirrels (Morton et al. 1974). Squirrels from late litters were smaller and weighed less than early born individuals, but in proportion, had similar fat reserves compared with the bigger early born juveniles at hibernation onset. In this study, this was, however, not the case for late-born garden dormice. Their pre-hibernation fat reserves, even as a proportion of BM, were significantly lower than in early born individuals. Hence, such lower pre-hibernation fat content could have important fitness consequences. Still, as shown in common dormice from a population also producing 2 l per year, no significant differences in winter survival between early and late-born juveniles of the same sex were detected (Bieber et al. 2012). However, in contrast to garden dormice, early born common dormice are able to reproduce in the year of their birth, prior to their first hibernation. Hence, further studies are clearly needed to determine long-term fitness consequences of being born late in free-ranging garden dormice.

### Differences in the timing of growth and fattening prior to winter hibernation

In our study, young individuals from early and late-born litters gained BM at similar rates from birth to the early weaning phase (~35 days post-birth). Furthermore, at early weaning time, juveniles had very low fat content (<6 %). Taken together, these results suggest that young garden dormice do not accumulate significant fat reserves, but mainly grow during the pre-weaning period. Growing fast is particularly important for hibernating species because of a short time between birth and winter immergence. For instance, milk from Colombian ground squirrels’ mothers contains relatively low amount of lipids but high protein and calcium contents (Skibiel and Hood 2013). Furthermore, milk protein amount was higher in Colombian ground squirrels than in other rodents, indicating the importance of fast growth rates for hibernating species (Skibiel and Hood 2013).

Whereas late-born juveniles showed a constant rate of growth and BM gain from the early weaning phase to their maximal body size, early born individuals slowed down these processes approximately 6 weeks after the start of the experiments. This phenomenon occurred independently of fluctuations in ambient temperature. Similar temporal patterns were already reported for Belding ground squirrels, in which the rate of BM gain was slowed down or even arrested at mid-season, followed by a phase of rapid BM gain prior to hibernation (Morton et al. 1974). Conversely to early born juveniles, individuals born late had to simultaneously ensure processes of growth and fattening, leading to a continuous BM gain.

In this study, male and female garden dormice differed significantly in the time they reached their maximum levels of BM and body size. Both early and late-born females entered hibernation 2 weeks earlier than males. This sex difference in the onset of hibernation was also observed in juvenile Belding ground squirrels, which might indicate a greater dispersal probability of male juveniles prior to hibernation (Morton et al. 1974). Alternatively, males might delay hibernation because of the sexual dimorphism that seems to occur in adult garden dormice, as previously discussed. Hence, males may stay active longer to reach larger body sizes compared with females prior to hibernation. This was indeed the case for early born males, which reached larger body sizes than females, but not for late-born males that showed structural sizes similar to early and late-born females. Hence, even by delaying hibernation, late-born juvenile males did not have sufficient time to reach a structural size similar to early born males.

## Conclusion

We conclude that late-born juvenile garden dormice grow structurally and gain BM almost twice as fast as early born individuals prior to their first winter hibernation. During lactation, young individuals born from both early and late litters mainly grow structurally and at similar rates. Growth acceleration among late born young occurs predominantly during the post-weaning period. Late-born females achieve similar levels of body size, but lower pre-hibernation BM, hence fat reserves, compared with early born individuals. Conversely, late-born males show both lower maximal body size and body FM, compared with their early born counterparts, prior to hibernation. Further studies would need to investigate potential negative long-term consequences of faster growth rates and/or suboptimal body size in late-born individuals in terms of reproductive success, aging processes, and lifespan.

## Footnotes

1 See also the section below for the relationship between body mass and pre-hibernation fattening.
